# *KIT* and *PDGFRA* Variants and the Survival of Patients with Gastrointestinal Stromal Tumor Treated with Adjuvant Imatinib

**DOI:** 10.3390/cancers15153879

**Published:** 2023-07-30

**Authors:** Heikki Joensuu

**Affiliations:** Department of Oncology, Helsinki University Hospital and University of Helsinki, 00029 Helsinki, Finland; heikki.joensuu@hus.fi; Tel.: +358-9-471-73200

**Keywords:** gastrointestinal stromal tumor, GIST, imatinib, adjuvant therapy, molecular targeted therapy, KIT, platelet-derived growth factor receptor alpha, survival

## Abstract

**Simple Summary:**

Gastrointestinal stromal tumor (GIST) is the most common type of sarcoma that arises from the gastrointestinal tract, most frequently from the stomach. The malignancy potential of GISTs varies greatly; some are aggressive cancers. Most localized GISTs are cured with surgery. Recent results show that adjuvant imatinib substantially improves the recurrence-free survival and overall survival of GIST patients who have a high risk for recurrence after surgery if GIST harbors an imatinib-sensitive mutation in *KIT* or *PDGFRA* and adjuvant imatinib is administered long enough after surgery, currently for 3 years.

**Abstract:**

Adjuvant imatinib improves the recurrence-free survival and overall survival (OS) of patients with gastrointestinal stromal tumors (GISTs) who have a high risk of recurrence after surgery and is now considered standard treatment. Yet, OS benefit has been demonstrated in only one randomized study, the Scandinavian Sarcoma Group XVIII/AIO trial, where patients with high-risk GISTs were allocated to either 1 year or 3 years of adjuvant imatinib. SSGXVIII/AIO is also the only randomized trial in which adjuvant imatinib duration exceeding 2 years was evaluated. In this trial, the 3-year treatment led to a 45% reduction in the risk of death during the first 10 years that followed random allocation even though some of the patients did not have GISTs at tumor histology review, had mutations now known to be imatinib-resistant or had non-localized disease at study entry. In the subgroup of patients who had *KIT* exon 11 deletion/indel mutation, the reduction in the risk of death was 66% in favor of the longer treatment. Proper patient selection is of crucial importance since many patients are cured with surgery. Little evidence for OS benefit is available from randomized trials for patients whose GIST harbors *KIT* exon 9 mutation, *KIT* insertion mutation, *PDGFRA* D842V mutation, or lacks *KIT* and *PDGFRA* mutations. Adjuvant imatinib improves OS substantially if high-risk GISTs can be identified, treatment duration is long enough, and GISTs harbor an imatinib-sensitive mutation.

## 1. Introduction

Most gastrointestinal stromal tumors (GISTs) are sporadic, and no risk factors have been identified for GISTs apart from a few rare tumor syndromes [1,2]. Patients with GISTs often present with bleeding into the bowel lumen or the abdominal cavity, but the symptoms vary, and some GISTs are asymptomatic when detected at abdominal imaging, surgery for other conditions, or via palpation. The imaging of the abdomen with or without endoscopy is essential in the diagnostic workup, but the diagnosis is made with immunohistochemistry from a biopsy or the resected tumor [2].

The clinical behavior of GISTs is highly variable, ranging from indolent tumors to aggressive cancers that give rise to metastases. At the more benign end of the spectrum, small, <1 cm size mini-GISTs can be found in the gastrointestinal tract of about 30% of the middle-aged and elderly general population. Mini-GISTs have little malignancy potential, although they may harbor similar *KIT* and *PDGFRA* (*platelet-derived growth factor receptor-alpha*) variants as larger, clinically detected GISTs [3,4]. Most (about 80%) clinically detected GISTs are localized at the time of detection [5]. About 60% of localized GISTs are cured via the surgical removal of the tumor. Metastases are usually located within the abdominal cavity or in the liver and are usually detected within the first 5 years following surgery and the great majority within the first 10 years [6].

About 75% of GISTs have an activating mutation in the *KIT* proto-oncogene, about 10–15% have an activating mutation in *PDGFRA*, and about 10% do not have a mutation in these genes [7,8]. *KIT* and *PDGFRA* encode the respective receptor tyrosine kinases, and they are considered important drivers of GIST molecular pathogenesis and excellent molecular targets for tyrosine kinase inhibitors [1]. The most common type of mutations in GISTs are *KIT* exon 11 mutations (about 65%), most of which are deletion or indel mutations, but substitution mutations and insertion/duplication mutations also occur in *KIT* exon 11. About 6–8% of localized GISTs harbor *KIT* exon 9 mutations. *KIT*-exon-9-mutated GISTs usually arise from the small intestine, unlike *KIT*-exon-11-mutated GISTs, which can occur anywhere along the gastrointestinal tract, and *PDGFRA*-mutated GISTs, which usually arise from the stomach [8]. *KIT*-exon-9-mutated GISTs tend to have a more aggressive clinical course than *KIT*-exon-11-mutated GISTs, whereas patients with *PDGFRA*-mutated GIST usually have a favorable prognosis and seldom give rise to metastases [8].

Imatinib, an orally administered selective tyrosine kinase inhibitor that inhibits *KIT*, *PDGFRA*, and a few other kinases [9], has been the standard first-line treatment for advanced GISTs for about two decades [10]. About 85% of GISTs either respond to imatinib administered 400 mg/day orally or stabilize, and most responses are durable [11,12]. Moreover, most patients tolerate imatinib well. The most common adverse effects are usually mild and include anemia, eyelid edema, diarrhea, and muscle cramps. Unfortunately, an overwhelming majority of GISTs eventually acquire mutations that lead to imatinib resistance [13]. The median time to disease progression on imatinib in advanced GISTs is 2–3 years [11]. Tyrosine kinase inhibitors sunitinib, regorafenib, and ripretinib are effective and approved as second-line, third-line, and fourth-line treatments, respectively, but the response durations achieved are usually substantially shorter than with first-line imatinib [14,15,16]. The median overall survival (OS) of patients with advanced GISTs is about 5 years [11]. In general, GISTs with *KIT* exon 11 mutations are sensitive to imatinib, and a durable response is often achieved, whereas *KIT* exon 9 mutations show less sensitivity, and some mutations, notably the *PDGFRA* exon 18 D842V mutation, are imatinib-insensitive [17,18,19,20]. Most patients with *PDGFRA* D842V mutation respond to another tyrosine kinase inhibitor, avapritinib [21].

Since advanced GIST is still usually a fatal disease, an important strategy to improve survival is to administer imatinib as an adjuvant or neoadjuvant treatment. To date, imatinib is the only agent that has been evaluated as an adjuvant treatment for GISTs. The US National Comprehensive Cancer Network (NCCN) now recommends the administration of adjuvant imatinib for at least 3 years after surgery to patients with a significant risk of GIST recurrence [22], and the European Society for Clinical Oncology (ESMO) recommends the treatment for 3 years [23]. There is, however, uncertainty about how to best select the patients for adjuvant imatinib, the optimal duration of adjuvant therapy, and the influence of *KIT* and *PDGFRA* mutational types on administering the treatment. This review focuses on these aspects of adjuvant therapy for GISTs. The neoadjuvant treatment of GISTs has been the topic of other recent reviews [24,25].

## 2. Identification of Patients with High-Risk GISTs

The patients who are likely cured with surgery need to be identified to avoid overtreatment with adjuvant therapy. Many prognostic factors have been described in GISTs, but the most important factors suggestive of cure with surgery alone are a low tumor mitotic count, small size, site of origin in the stomach, and the absence of tumor rupture [26]. Since none of these four factors is sufficient for prognostication alone, several multifactorial risk stratification tools have been developed. Such tools include risk stratification tables, nomograms, genetic classifiers, and a prognostic heat map [26]. Of these, the National Institutes of Health (NIH) consensus criteria [27], the modified NIH scheme [28], and the Armed Forces Institute of Pathology (AFIP) classification [29] are probably the most frequently used and the most relevant for this review.

The NIH Consensus criteria estimate the risk of GIST recurrence after surgery using tumor diameter and mitotic count, the AFIP criteria consider tumor site in the gastrointestinal tract as the third factor, and the modified NIH criteria also consider tumor rupture, thus encompassing all four factors. The performance of these three risk stratification schemes has been validated and found to be approximately similar [6,30]. The NIH consensus classification and the modified NIH classification stratify GISTs into four prognostic categories (very low risk, low risk, intermediate risk, and high risk), and the AFIP classification classifies the disease into eight categories (1, 2, 3a, 3b, 4, 5, 6a, and 6b). From a practical point of view, the main difference between the NIH consensus classification and the modified NIH classification is that the modified criteria provide a better distinction between intermediate- and high-risk patients, the intermediate-risk patients having roughly a similar risk for recurrence as patients at low or very low risk of GISTs. The AFIP criteria result in a spectrum of gradually increasing risk from very good (group 1) to very poor (group 6b) [6].

All three commonly used prognostication schemes have limitations. One limitation is that they categorize continuous variables, with tumor size using three cut-off values (2 cm, 5 cm, and 10 cm) and mitotic count with either one cut-off (the AFIP scheme, 5 mitoses/50 high-power fields of the microscope) or two cut-offs (the other two schemes, 5 and 10 mitoses). This may result in widely different estimations for the risk of recurrence even for tumors with only a slight difference in size or the mitotic count when these parameters are close to the cut-off value, and thus may lead to opposite decisions regarding adjuvant treatment. The use of prognostication tools where the mitotic count and size are treated as continuous variables makes more sense [6], but their performance has not been tested in prospective clinical trials. Another limitation is that the high-power field-of-view size of the microscopes may vary and has increased with time, making the calculation of the number of mitoses per 5 mm^2^ recommendable compared with high-power fields. Neoadjuvant imatinib often radically reduces the mitotic count and tumor size, making prognostication from the residual tumor tissue challenging [31].

Prognostication methods likely need refinement. GIST mutational types provide some further guidance since some mutations such as *KIT* exon 11 deletion mutations at 557–558 tend to be associated with high mitotic counts, whereas *PDGFRA* mutations are associated with low counts, but the mitotic counts associated with each mutation may vary widely [6]. Many studies have focused on specific features in GIST biology, some of which seem promising regarding the refinement of prognostication [32].

## 3. Randomized Trials Evaluating Adjuvant Imatinib

Three registered randomized phase 3 trials and four non-randomized phase 2 trials have evaluated imatinib as an adjuvant treatment for patients with GISTs (Table 1 and Table 2) [25]. All studies were open-label, and the starting dose of imatinib was 400 mg/day orally [33,34,35,36,37,38,39,40,41,42]. The primary endpoint was recurrence-free survival (RFS) except for two studies [33,34,35]. Mutation analysis for *KIT* and *PDGFRA* was carried out in most studies.

The ACOSOG Z9001 trial compared adjuvant imatinib to placebo, each administered for 12 months after macroscopically complete surgery for localized GISTs. Patients whose GIST was ≥ 3 cm in diameter were eligible regardless of the other features of the tumor. After a median follow-up time of 19.4 months, the 1-year RFS was 98% in the imatinib group and 83% in the placebo group (hazard ratio (HR): 0.35, 95% confidence interval (CI): 0.22–0.53, *p* < 0.0001) [36]. There was no difference in OS between the groups at the time of the trial reporting. When the trial was reanalyzed after a longer follow-up time of 74 months for the subset of 645 patients with mutation analysis results and mitotic counts available, RFS still favored the imatinib group (HR: 0.6; 95% CI: 0.43–0.75; *p* < 0.001) [37].

The EORTC/Intergroup trial compared 2 years of adjuvant imatinib to observation in a patient group with intermediate- or high-risk GISTs according to the NIH consensus classification [34,35]. The primary endpoint was originally OS but was changed to imatinib monotherapy failure-free survival (IFFS) during the study due to improvement in the prognosis of patients with advanced GISTs. IFFS was defined as the time from the date of randomization to the date of the start of a second systemic treatment or death. In the final analysis, carried out after a median follow-up of 9.1 years, there was no difference between the imatinib and observation arms in IFFS or OS, but RFS was significantly better in the imatinib group (5-year RFS 70% vs. 63%, respectively; HR: 0.71; 95% CI: 0.57–0.89) [35].

Unlike the ACOSOG Z9001 and EORTC/Intergroup trials, the Scandinavian Sarcoma Group (SSG) XVIII/German (AIO) trial accrued only patients with a high estimated risk for recurrence despite macroscopically complete open surgery [38,39]. The risk was estimated using the modified NIH scheme [28]. Of the 400 patients accrued, 200 patients were randomly assigned to receive adjuvant imatinib 400 mg/day orally for 1 year, and the remaining 200 patients received it for 3 years. After a median follow-up of 9.9 years, patients allocated to the 3-year group had longer RFS than those assigned to the 1-year group (10-year RFS: 52.5% vs. 41.8%; HR: 0.66; 95% CI: 0.49–0.87; *p* = 0.003), and OS was also longer in the 3-year group (10-year OS: 79.0% vs. 65.3%; HR: 0.55; 95% CI: 0.37–0.83) [39]. This analysis also included 15 patients who did not have GISTs in histological review and 24 patients who had metastatic disease at the time of randomization. When these patients were excluded from the analysis, the 10-year RFS was 52.4% in the 3-year group and 44.2% in the 1-year group (HR: 0.70; 95% CI: 0.52–0.94; *p* = 0.02), and the 10-year OS rates were 81.6% and 66.8%, respectively (HR: 0.50; 95% CI: 0.32–0.80; *p* = 0.003).

## 4. Adjuvant Imatinib and Overall Survival

While RFS improved compared with the control group in all three randomized phase 3 trials, only the SSGXVIII/AIO trial found adjuvant imatinib to improve OS. In the SSGXVIII/AIO trial, the improvement in OS was substantial, since in the intention-to-treat population, 45% of deaths were avoided in the 3-year group compared with the 1-year group during the 10-year follow-up.

A relevant question is why an OS benefit was observed in the SSGXVIII/AIO trial but not in the other two randomized trials. SSGXVIII/AIO was the only trial where a relatively long duration of adjuvant imatinib was evaluated, and it could be argued that adjuvant imatinib needs to be administered at least for 3 years to impact OS. Yet, since the control group patients in the SSGXVIII/AIO trial were treated with adjuvant imatinib for 1 year, the difference in the duration of imatinib administration between the two study arms was 2 years as was the case also the EORTC/Intergroup trial, where no significant difference in OS was observed between the allocation groups. One could speculate that >2-year durations of imatinib administration could have a greater biological effect on occult GIST deposits, reducing their malignancy potential more than shorter durations, but little evidence is available to support this hypothesis.

The three trials had different patient eligibility criteria, which likely influenced the survival outcomes. Patients cured with surgery are only harmed with adjuvant imatinib, and the greater the proportion of such patients, the more difficult it is to demonstrate a survival benefit from adjuvant therapy. The key eligibility criterion in ACOSOG Z9001 was tumor diameter ≥3 cm, but a substantial proportion of such patients are cured with surgery [6,37]. The relatively short median follow-up time, a limited number of OS events, and allowing cross-over from the placebo arm to the imatinib arm in the event of tumor recurrence may also have impaired the assessment of OS in the first analysis of the trial [36]. In the second analysis of the trial that focused on the subset of patients with tumor specimens available (n = 645, 90.5% of the 713 patients randomized), RFS favored the imatinib group after a median follow-up of 74 months (HR: 0.6; 95% CI: 0.43–0.75; *p* < 0.001), but there was no difference in OS between the two arms [37].

Patients with an intermediate risk of recurrence were allowed to enter the EORTC/Intergroup trial, unlike the SSGXVIII/AIO trial. A substantial majority of intermediate-risk GIST patients are cured with surgery regardless of whether the NIH Consensus criteria or the modified NIH criteria are used to define the risk [6]. When the patients were stratified by risk group, the 10-year IFFS and OS survival rates were high (≥90%) in the intermediate-risk subgroup, and the Kaplan–Meier plots for IFFS and OS overlapped between the 2-year imatinib group and the observation group, whereas in the high-risk subgroup, IFFS and OS slightly favored the imatinib arm [35]. About 52% of the trial patients had low-/intermediate-risk GISTs, which may have reduced the trial power to detect a statistical difference in OS. 

Among the four registered non-randomized trials that have evaluated adjuvant imatinib, a substantially higher 5-year RFS rate of about 90% was achieved in trials that evaluated either 3-year [40] or 5-year [41] adjuvant imatinib compared with the two trials where the patients were scheduled for 1-year of adjuvant imatinib [33,42] (Table 2). Yet, intermediate-risk patients were allowed to participate in the trials that evaluated 3- or 5-year adjuvant imatinib, unlike the trials that tested 1-year imatinib, which likely improved the survival rates and makes efficacy comparisons between the trials challenging (Table 2).

In summary, the results from the three randomized trials indicate that adjuvant imatinib improves RFS. Adjuvant imatinib administered for 3 years substantially improved OS compared with 1-year administration in the SSGXVIII/AIO trial, which focused on high-risk patients who likely benefit most from adjuvant imatinib. Patients considered for adjuvant imatinib need to be identified using one of the validated multiparameter prognostication schemes to avoid overtreatment or undertreatment. There may not be large differences between the most often used risk stratification schemes with this respect, but at present, an OS benefit has been demonstrated only when the modified NIH selection criteria were used for the detection of high-risk GISTs. 

## 5. *KIT* and *PDGFRA* Mutational Types in Patient Selection for Adjuvant Treatment

*KIT* and *PDGFRA* mutational genotypes are associated with prognosis in patients treated with adjuvant imatinib. In general, patients with *KIT* exon 9 mutations have unfavorable prognosis compared with patients with *KIT* exon 11 deletion/indel mutation, whereas patients with *KIT* exon 11 insertion/duplication mutation and those with *PDGFRA* mutation tend to have the most favorable survival [33,37,43]. The frequencies of the main *KIT* and *PDGFRA* mutational types are relatively similar across the registered studies conducted (Table 3). The proportion of patients with *KIT* exon 9 mutation was the highest (12.8%) in the ACOSOG Z9000 patient population and the lowest in the PERSIST-5 trial population (3.5%), an observation that is compatible with the high 5-year RFS achieved in PERSIST-5 (Table 2).

### 5.1. KIT Exon 11 Deletion and Indel Variants

*KIT* exon 11 deletion/indel variants are the most common type of *KIT* mutations, occurring in about 40% of unselected GISTs [8] and adjuvant trial participants with data available (Table 3). *KIT* exon 11 deletion and indel variants are sensitive to imatinib in vitro [17,44], and patients with *KIT* exon 11 deletion mutation treated with imatinib in the metastatic setting benefit more from imatinib than patients with other types of *KIT* mutation [17,18,44]. 

Patients with *KIT* exon 11 deletion variants also benefit most from adjuvant imatinib. In the ACOSOG Z9001 trial patient population, 1-year adjuvant imatinib significantly improved RFS, compared with placebo in the subset with *KIT* exon 11 deletion mutation, whereas a similar improvement in RFS was not observed in the subsets of patients with *KIT* exon 11 substitution mutation, *KIT* exon 11 insertion mutation, or *KIT* exon 9 mutation [37]. 

In the SSGXVIII trial patient population, patients with *KIT* exon 11 deletion/indel mutation substantially benefitted from the 3-year duration of adjuvant imatinib compared with 1-year imatinib, with 52% reduction in the risk of GIST recurrence and 66% reduction in the risk of death [45]. The 10-year RFS was 47% in the 3-year group and 29% in the 1-year group (HR: 0.48; 95% CI: 0.31–0.74; *p* < 0.001), and the 10-year OS rates were 86% and 64%, respectively (HR: 0.34; 95% CI: 0.15–0.72; *p* = 0.007). This impressive 66% reduction in the risk of death during a median patient follow-up of 10 years may still underestimate imatinib efficacy since the control group patients also received imatinib.

### 5.2. KIT Exon 11 Substitution Variants

*KIT* exon 11 substitution variants occur in 20–25% of unselected GISTs [7,8], and they also occurred at a similar frequency in the adjuvant trials (Table 3). Most *KIT* exon 11 substitution variants are imatinib-sensitive in vitro, and most patients with advanced GISTs with *KIT* exon 11 substitution variants respond to imatinib [44].

Relatively few data are available from clinical trials about the survival benefit of adjuvant imatinib in the subset of patients with the *KIT* exon 11 substitution variant. In the ACOSOG Z9001 trial, there was no difference in RSF between the 57 patients assigned to 1-year imatinib and the 54 patients assigned to placebo (*p* = 0.96) [37]. In the SSGXVIII/AIO trial, the 10-year RFS and OS rates numerically favored the 3-year arm in a subset of 68 patients with *KIT* exon 11 substitution mutation (10-year RFS 64% vs. 54%; 10-year OS 81% vs. 66%), but no statistically significant benefit could be demonstrated from 3-year adjuvant imatinib compared with 1-year imatinib either in RFS or OS [45]. These subgroup analyses need to be viewed with caution because of the relatively small patient and event numbers.

In general, patients with the *KIT* exon 11 substitution variant are recommended to be treated with adjuvant imatinib even though the magnitude of the benefit remains unknown. The survival benefit for patients with these variants may be more limited than for those with *KIT* exon 11 deletion variants (Table 4).

### 5.3. KIT Exon 11 Insertion/Duplication Variants

*KIT* exon 11 insertion/duplication variants are relatively rare. They occur in 2–4% of clinical GISTs [7,8], and 6–9% of the patients who participated in registered adjuvant trials had the *KIT* exon 11 insertion variant (Table 3). Few data are available about their sensitivity to imatinib either in vitro or in silico, but they appear to be sensitive to imatinib in vivo [44].

The patients with *KIT* exon 11 insertion/duplication mutation had about 80% 5-year RFS rate in the ACOSOG Z9001 trial [37] (*n* = 46) and a 75% rate in the SSGXVIII/AIO trial [45] (*n* = 22). The 10-year OS rate was high (about 90%) in the SSGXVIII trial. There was no difference in RFS between the 1-year imatinib group and the 1-year placebo group in the ACOSOG Z9001 trial, and no difference in RFS or OS was observed between the 3-year group and the 1-year group in the SSGXVIII/AIO trial, but these analyses are hampered by small patient and event numbers.

### 5.4. KIT Exon 9 Variants

*KIT* exon 9 variants occur in about 6% of unselected GISTs [7,8] and about 10% of GISTs treated with adjuvant imatinib (Table 3). Almost all *KIT* exon 9 variants are Ala502_Tyr503 duplications. Patients with the *KIT* exon 9 variant have less favorable RFS and OS when treated with adjuvant imatinib than patients with other types of mutations [33,45]. *KIT*-exon-9-mutated GISTs are imatinib-sensitive in vitro/in silico, but higher drug doses may be needed [44]. A subgroup analysis from merged data from two large randomized trials that compared an imatinib dose of 400 mg once daily with 400 mg b.i.d. in patients with overtly metastatic GISTs indicated that, unlike patients with other types of mutations, patients with *KIT* exon 9 mutations achieved longer progression-free survival when treated with the 800 mg/day dose [20]. This observation suggests that patients treated with the standard 400 mg/day dose in the adjuvant setting could be underdosed. A recent retrospective non-randomized cohort study comprising 185 patients with *KIT* exon 9 mutations and treated with adjuvant imatinib challenged this view, since in this series, the 800 mg/day dose was not associated with longer RFS than the standard 400 mg/day dose [47].

In the ACOSOG Z9001 trial, 1-year adjuvant imatinib 400 mg/day had similar efficacy as placebo on RFS in the subset of 35 patients with *KIT* exon 9 mutations (*p* = 0.87) [37]. Similarly, 3-year adjuvant imatinib conferred no RFS or OS benefit compared with 1-year imatinib in this mutational subset consisting of 26 patients in the SSGXVIII/AIO trial [45]. Since the numbers of patients in these subgroup analyses are small for drawing conclusions about imatinib efficacy, the survival benefit of adjuvant imatinib in patients with *KIT* exon 9 mutations remains unknown (Table 4). One option is to treat patients with *KIT* exon 9 mutations with adjuvant imatinib using a ≥ 400 mg/day dose for 3 years, perhaps tailoring the dose with treatment tolerability.

### 5.5. PDGFRA Mutations

About 10–15% of GISTs harbor a mutation in *PDGFRA*, of which more than half are exon 18 D842V substitution mutations [7,8]. The *PDGFRA* D842V variant is insensitive to imatinib in vitro and in vivo [19,44] but sensitive to avapritinib [21,48]. Most of the other mutations that occur in *PDGFRA* exon 18 or *PDGFRA* exons 5, 12, and 14 are imatinib-sensitive [44]. *PDGFRA*-mutated GISTs usually arise from the stomach and have a low mitotic count and favorable prognosis [8,33,45].

In the SSGXVIIII/AIO trial patient population with *PDGFRA* D842V mutation (*n* = 30), adjuvant imatinib duration did not influence RFS or OS. The 10-year RFS and OS were high (about 80% and 90%, respectively), although the tumors had high-risk features, and there was likely no benefit from adjuvant imatinib [45]. This observation suggests that the current risk-stratification criteria may overestimate the recurrence risk associated with D842V-mutated GISTs, potentially leading to overtreatment with adjuvant or neoadjuvant therapies.

Patients with *PDGFRA* D842V mutations are not recommended to be treated with any duration of adjuvant imatinib [23] (Table 4). The NCCN guidelines [22], but not the ESMO guidelines [23], suggest considering neoadjuvant avapritinib, but only if morbidity related to surgery could be reduced through tumor downsizing. The NCCN guidelines note that neoadjuvant therapy may prohibit an accurate assessment of the risk of recurrence after tumor resection. No clinical trial has evaluated neoadjuvant or adjuvant avapritinib.

### 5.6. GISTS with No KIT Or PDGFRA Mutation

GISTs with neither *KIT* nor *PDGFRA* mutation, previously called “wild-type GISTs”, are a heterogeneous group of tumors that predominantly occur in children or young females and may harbor several types of molecular aberrations [49]. None of these aberrations, which include *SDH* (encodes succinate dehydrogenase) mutations, epigenetic silencing of the *SDHC* promoter, and *NF1* (encodes neurofibromatosis-1 protein) and *BRAF* mutations, is imatinib-sensitive. Some non-*KIT*/non-*PDGFRA* GISTs progress very slowly, sometimes over a few decades.

Non-*KIT*/non-*PDGFRA* GISTs constitute about 10% of all GISTs [7,8,49], but the true incidence may be <10%, since their frequency may be inflated by GISTs that harbor an undetected *KIT* or *PDGFRA* mutation [46]. Mutations that are challenging to detect in sequencing include deletions at the *KIT* intron 10/exon 11 boundary and large insertions and deletions that exceed 24 base pairs [46]. Therefore, a repeat mutation analysis, perhaps with a different technique from the one used in the first analysis, should be considered at a low threshold to avoid missing an imatinib-sensitive mutation. The repeat analysis is particularly worthwhile when the patient does not have typical clinical features suggestive of syndromic or familial GISTs, such as young age, female gender, gastric location, or presence of lymph node metastases. Neurofibomatosis-1-associated GISTs are often multiple and located in the jejunum [50].

The RFS of patients with non-*KIT-*/non-*PDGFRA*-mutated GISTs was not improved with 1-year adjuvant imatinib in the ACOSOG Z9001 trial [37], and 3-year adjuvant imatinib did not significantly improve RFS or OS compared with 1-year imatinib in the SSGXVIII/AIO trial [45], but the number of events in these analyses was small. GISTs with neither *KIT* nor *PDGFRA* mutation are not recommended to be treated with adjuvant imatinib (Table 4).

## 6. Influence of Adjuvant Imatinib on Imatinib Efficacy after GIST Recurrence

One of the most important questions related to adjuvant imatinib is whether drug resistance may develop during adjuvant imatinib and whether this could impair imatinib efficacy when imatinib is restarted for the treatment of recurred GISTs. To study this hypothesis, the investigators of the EORTC/Intergroup trial chose to use IFFS, the time from the date of randomization to imatinib monotherapy failure, as the primary endpoint of the trial [34,35]. Imatinib failure usually occurs when drug resistance develops while the patient is being treated for advanced GIST. There are two prerequisites for selecting IFFS as the endpoint: the first is that imatinib should be selected as the first-line agent once GIST recurs after adjuvant imatinib, and the second is that adjuvant imatinib is usually not curative. In the EORTC/Intergroup trial, there was no significant difference in IFFS between the 2-year imatinib arm and the observation arm (Table 1).

GIST recurrence is relatively rare while the patient is receiving adjuvant imatinib and occurs in about 5% of the patients [33,39,40,41,42]. Some of the patients who recur on adjuvant imatinib have imatinib-insensitive mutations such as *PDGRFA* D842V [41] or non-*KIT*/non-*PDGFRA* GIST [42]. Patients with *KIT* exon 9 mutations have GIST recurrence more frequently on adjuvant imatinib 400 mg/day than patients with *KIT* exon 11 mutations. In the SSGXVIII/AIO trial, 7 (4.7%) of the 149 patients with *KIT* exon 11 mutations had GIST recurrence while the patient was on imatinib, whereas 6 (23.1%) of the 26 patients with *KIT* exon 9 mutations had recurrence during imatinib treatment [45].

The recent results from the SSGXVIII/AIO trial suggest that adjuvant imatinib does not have a major influence on imatinib efficacy in an advanced setting and may not lead to the earlier emergence of imatinib resistance. In the SSGXVIII/AIO trial population, most patients were treated with first-line imatinib when GIST had recurred, and in the subset of patients whose GIST recurred, the median duration of OS calculated from the date of GIST recurrence to the date of death or the date of the last follow-up was similar in the 3-year group and the 1-year group (6.4 vs. 6.7 years, respectively; HR: 1.00; 95% CI; 0.62–1.61, *p*  > 0.99) [39]. This observation suggests that the longer adjuvant imatinib administration did not influence the efficacy of first-line imatinib or other tyrosine kinase inhibitors used in the later lines to treat advanced GISTs.

The over 6-year median OS times achieved in both arms of the SSGXVIII trial once GISTs had recurred as overtly metastatic disease compare well with the median survival time of about 5 years found in patient populations with newly detected overtly metastatic GISTs and where the patients were not treated with adjuvant imatinib [11]. The over 6-year median OS achieved in the metastatic setting in the SSGXVIII/AIO trial population could also lend support to the hypothesis that adjuvant imatinib does not have a major influence on imatinib efficacy should GIST recur, but comparisons between studies need to be interpreted with caution due to different patient populations. The SSGXVIII/AIO trial participants were followed up with longitudinal imaging using either computed tomography or MRI, which usually led to the early detection of recurrence at an asymptomatic stage when the tumor volume was still small. The early detection of metastases causes a lead time bias favoring patients followed up with imaging compared with patients whose metastases are larger and symptomatic when detected. Furthermore, the detection of metastases when the tumor volume is still small likely decreases the risk of early emergence of acquired drug resistance mutations, since the likelihood of such mutations is probably lower when there is a smaller number of tumor cells.

Taken together, the available data suggest that the development of acquired drug resistance during adjuvant imatinib is infrequent and that prior adjuvant imatinib does not markedly impair imatinib efficacy for overtly metastatic GIST should GIST recur after adjuvant imatinib. In the SSGXVIII/AIO trial, the substantial OS benefit in favor of the 3-year group resulted from the longer adjuvant treatment and not from the subsequent treatments administered for overtly metastatic GIST. This may imply that a failure to offer adjuvant imatinib to a high-risk GIST patient cannot be compensated later by the greater efficacy of imatinib administered for advanced disease.

## 7. Adjuvant Imatinib Treatments Longer Than Three Years

Some GIST patients might benefit from longer than 3-year administration of adjuvant imatinib. This hypothesis is supported by the shape of the Kaplan–Meier plots depicting RFS in the SSGXVIII/AIO and EORTC/Intergroup trials, where a substantial drop in RFS occurred within the 1–2 years following the discontinuation of adjuvant imatinib, indicating that many GISTs progressed quickly after adjuvant imatinib was discontinued [35,39]. In the SSGXVIII/AIO trial, the abrupt drop in RFS was greater in the group of patients treated with 1-year imatinib than in the 3-year group, and it was most evident in the subset of patients with *KIT* exon 11 deletion or indel mutations, suggesting that patients with imatinib-sensitive mutations might particularly benefit from over 3-year duration of adjuvant imatinib treatment [45].

To date, no registered randomized trial has reported results from adjuvant imatinib treatments lasting longer than three years. One ongoing randomized phase 3 trial (ImadGIST, NCT02260505) with an accrual target of 134 patients is comparing six to three years of adjuvant imatinib treatment, and another phase 3 trial (SSGXXII, NCT02413736) with an accrual target of 250 patients is comparing five to three years of adjuvant imatinib treatment.

In the PERSIST-5 trial, a promising 5-year RFS rate of 90% and an OS rate of 95% were achieved with adjuvant imatinib scheduled for 5 years, but both intermediate- and high-risk patients were eligible. Half (49%) of the patients discontinued imatinib early, most commonly due to patient choice [41]. A few unregistered cohort studies have compared 5-year adjuvant imatinib to shorter durations of imatinib treatment. Some of these studies suggest that higher RFS can be achieved by administering imatinib for longer than 3 years compared with 3 years [51,52]. An example is an observational registry study from Japan, where 515 patients with high-risk GISTs were treated with 3-year administration of adjuvant imatinib or >3-year administration of imatinib (median, 5 years) and prospectively followed up for a median of 5.9 years. In this study, patients treated for >3 years achieved longer adjusted RFS than patients treated for 3 years, but adjusted OS was similar between the groups [51].

Even life-long administration of adjuvant imatinib has been considered when the risk of recurrence is particularly high. Patients with tumor rupture face a very high risk of recurrence, which has in some studies approached 100% [53]. Patients with rupture might, therefore, be comparable to patients with metastatic GISTs [23,54]. There are, however, different types of GIST rupture, and only ruptures classified as major are associated with a very high risk of recurrence, whereas a tumor mucosal defect or an intraluminal bowel perforation should probably not be considered ruptures [54,55]. Not all patients who were reported to have tumor rupture had recurrence during the follow-up in the trials addressing adjuvant imatinib [34,56]. Besides tumor rupture, the risk of GIST recurrence is also very high in the subset of patients with large, non-gastric GISTs with a very high mitotic count [6].

The NCCN guidelines recommend three years or longer adjuvant imatinib for patients with a significant risk of recurrence, but the guidelines provide no recommendation about the treatment duration when it exceeds three years [22]. The potential survival benefits gained from longer than 3 years of treatment with adjuvant imatinib, if any, need to be balanced with longer-lasting adverse effects and financial toxicity. The ESMO Clinical Practice Guidelines recommendation to treat high-risk patients with 3 years of adjuvant imatinib administration is easy to defend [23], but this policy should be coupled with robust evaluations of longer than 3-year durations in clinical trials, particularly in the subset of patients with an imatinib-sensitive *KIT* or *PDGFRA* variant.

## 8. Adjuvant Imatinib and Cure from GIST

Most lines of evidence from adjuvant trials suggest that adjuvant imatinib may only delay GIST progression and not be curative [34,35,36,37,51], but the recent results from the SSGXVIII/AIO trial based on a mature 10-year median follow-up raise the question of whether adjuvant imatinib could sometimes be curative. The key target group where adjuvant imatinib could most likely be curative are patients with an imatinib-sensitive mutation, such as patients with a *KIT* exon 11 deletion variant. In the SSGXVIII/AIO trial, the 102 patients with a *KIT* exon 11 deletion mutation (with no insertion component) had 47% 10-year RFS in the 3-year arm and 17% in the 1-year arm (HR: 0.34; 95% CI: 0.20–0.56; *p* < 0.001) and 83% 10-year OS in the 3-year arm and 59% in the 1-year arm (HR: 0.35; 95% CI: 0.14–0.81; *p* = 0.017) [45]. Since a great majority of GIST recurrences occur within the first 10 years of follow-up after surgery [6], the 30% difference in the 10-year RFS rate in favor of the 3-year group compared with the 1-year group could be suggestive of a curative effect in a fraction of patients. However, the administration of adjuvant imatinib may delay the progression of occult metastases, and even the 10-year median follow-up time may not be long enough for drawing definite conclusions. Unfortunately, the follow-up of the SSGXVIII/AIO trial participants has ended, and obtaining further follow-up data seems not feasible [39].

## 9. Conclusions

Adjuvant imatinib improved RFS in all three randomized phase 3 trials conducted, and 3-year adjuvant imatinib reduced the risk of death by 45% compared with 1-year imatinib in the SSGXVIII/AIO trial intention-to-treat population during a median patient follow-up of 10 years. This large improvement in OS in favor of the 3-year group occurred even though some of the trial participants had a tumor other than GIST at histology review, had a mutation now known to lead to imatinib insensitivity, or had non-localized disease already at study entry. There was an impressive 66% reduction in the risk of death in the subset of patients who had localized GISTs with an imatinib-sensitive *KIT* exon 11 deletion/indel mutation.

Adjuvant imatinib substantially improves OS, but only when the high-risk patients who may benefit from it can be reliably identified since most low- and intermediate-risk patients are cured with surgery and are harmed with adjuvant imatinib. This critically important task, i.e., the identification of high-risk patients, could be achieved successfully using the modified NIH criteria in the SSGXVIII/AIO trial. The optimal duration of adjuvant imatinib is unknown, but durations of less than 3 years may be suboptimal. The mutational analysis of GISTs is mandatory to exclude patients who have an imatinib-insensitive mutation or who lack mutation in *KIT* or *PDGFRA* from adjuvant treatment.

## 10. Future Directions

As with many other types of human cancer, improving OS with systemic adjuvant treatment is feasible in GISTs. The current standard of 3-year adjuvant imatinib treatment needs to be compared with longer than 3-year durations of imatinib administration, particularly in the subsets of patients with imatinib-sensitive mutations. This is already ongoing, but only two randomized trials are currently addressing the duration question.

Imatinib is generally well tolerated, generic, and highly effective, particularly for GISTs with *KIT* exon 11 deletion/indel mutations, thus raising the bar to be overcome by other agents that are approved for the treatment of advanced GISTs but not for the adjuvant setting. Avapritinib is highly effective for GISTs with *PDGFRA* D842V mutations but has not been approved for adjuvant treatment. Most D842V-mutated GISTs are cured with surgery.

The efficacy of adjuvant imatinib in the treatment of patients with *KIT* exon 9 mutations is unclear, and there is uncertainty about the optimal imatinib dose. Therefore, adjuvant trials focusing on the treatment of patients with *KIT* exon 9 mutations would be welcome. Focused studies on GISTs with *KIT* exon 11 insertion/duplication mutations also seem worthwhile to carry out.

The current risk stratification schemes work reasonably well but are not ideal in identifying the patients who are cured with surgery, leaving room for improvement. Patients participating in adjuvant trials should be followed up at least for 10 years, but preferably longer, to adequately address the influence of the interventions on OS. The current methods to follow up with patients during and after adjuvant therapy involve repeat imaging with associated costs and radiation hazards, and, therefore, blood-sampling-based methods for the early detection of recurrence and assessing the risk of recurrence would be highly welcome.

## Figures and Tables

**Table 1:** Randomized phase 3 multicenter trials evaluating adjuvant imatinib in GISTs.

Trial Name; No. of Accrued Patients	Investigational Group	Control Group	Patient Eligibility	Primary Endpoint	Mutation Analysis Available	Main Results
ACOSOG Z9001 (NCT00041197) *n* = 713 [36,37]	Imatinib 400 mg/d for 12 mo	Placebo for 12 mo	Size ≥ 3 cm	RFS	Yes	Median FU: 1.6 yrs 1-y RFS: IM, 98%; C, 83%; HR (CI): 0.35 (0.22–0.53) 1-y OS: IM, 99.2%; C, 99.7%; HR (CI): 0.66 (0.22–2.03)
EORTC/Intergroup (NCT00103168) *n* = 908 [34,35]	Imatinib 400 mg/d for 24 mo	Observation	Intermediate or high risk (NIH Consensus)	IFFS	No	Median FU: 9.1 yrs 5-y IFFS: IM, 87%; C, 83%; HR (CI): 0.87 (0.65–1.15) 5-y RFS: IM, 70%; C, 63%; HR (CI) 0.71 (0.57–0.89); 10-y RFS: IM, 63%; C, 61% 5-y OS: 5-y OS; IM, 93%; C, 92%; HR 0.88 (0.65–1.21); 10-y OS: IM, 80%, C, 78%
SSGXVIII (NCT00116935) *n* = 400 ^1^ [38,39]	Imatinib 400 mg/d for 3 yrs	Imatinib 400 mg/d for 1 yr	High risk (modified NIH)	RFS	Yes	Median FU: 9.9 yrs 10-y RFS: 3-y, 52.5%; C: 41.8%; HR (CI): 0.66 (0.49–0.87) 10-y OS: 3-y, 79.0%; C: 65.3%; HR (CI): 0.55 (0.37–0.83)

ACOSOG: American College of Surgeons Oncology Group; C: control group; CI: confidence interval; EORTC: European Organisation for Research and Treatment of Cancer; FU: follow-up; HR: hazard ratio: IFFS: imatinib monotherapy failure-free survival; IM: imatinib; NIH: National Institutes of Health; OS: overall survival; RFS: recurrence-free survival; SSG: Scandinavian Sarcoma Group. ^1^ Here, 3 of the 400 patients were randomized without the patient signing informed consent and were excluded.

**Table 2:** Registered prospective non-randomized phase 2 trials evaluating adjuvant imatinib in GISTs.

Trial Name; No. of Accrued Patients	Adjuvant Treatment	Patient Eligibility	Primary Endpoint	Mutation Analysis Available	Main Results
ACOSOG Z9000 (NCT00025246) *n* = 106 [33]	Imatinib 400 mg/d for 12 mo	High risk ^1^	OS	Yes	Median FU: 7.7 yrs 5-yr RFS 40%; 5-y OS 83%
Kanda et al. (NCT00171977) *n* = 64 [42]	Imatinib 400 mg/d for 12 mo	High risk (NIH Consensus)	RFS	Yes	Median FU: 2.1 yrs 3-yr RFS 57%; 3-y OS 87%
Li et al. (ChiCTR-TCC-00000582) *n* = 56 [40]	Imatinib 400 mg/d for 3 yrs	High or intermediate risk (NIH Consensus)	RFS	Yes ^2^	Median FU: 3.8 yrs 3-yr RFS 89%
PERSIST-5 (NCT00867113) *n* = 91 [41]	Imatinib 400 mg/d for 5 years	High or intermediate risk (AFIP)	RFS	Yes	Median FU: not provided 46 (50.5%) completed treatment 5-yr RFS 90%; 5-y OS 95%

ACOSOG: American College of Surgeons Oncology Group; AFIP: Armed Forces Institute of Pathology; FU: follow-up; NIH: National Institutes of Health; OS: overall survival; RFS: recurrence-free survival. ^1^ Size >10 cm, intraperitoneal tumor rupture, or ≤4 peritoneal implants; ^2^ limited data; mutation analysis available from 20 (35.7%) of the 56 patients.

**Table 3:** Frequency of *KIT* and *PDGFRA* mutational subsets in registered trials on adjuvant imatinib in GISTs.

*KIT/PDGFRA* Aberration	ACOSOG Z9001 *N* = 713 [36] *n* (%)	SSGXVIII/AIO *N* = 358 ^1^ [38] *n* (%)	ACOSOG Z9000 *N* = 106 [33] *n* (%)	Kanda et al. *N* = 64 [34] *n* (%)	PERSIST-5 *N* = 91 [41] *n* (%)
**Mutational data available**	507 (71.1)	341 (95.3)	78 (73.6)	62 (96.9)	85 (93.4)
***KIT***	386 (76.1)	273 (80.1)	59 (75.6)	55 (88.7)	64 (75.3)
*KIT* exon 11	341 (67.3)	239 (70.1)	45 (57.7)	49 (79.0)	57 (67.1)
Deletion/indel	184 (36.3)	149 (43.7)	N.P.	N.P.	N.P.
Substitution	111 (21.9)	68 (19.9)	N.P.	N.P.	N.P.
Insertion/duplication	46 (9.1)	22 (6.5)	N.P.	N.P.	N.P.
*KIT* exon 9	35 (6.9)	26 (7.6)	10 (12.8)	6 (9.7)	3 (3.5)
*KIT* other	10 (2.0)	8 (2.3)	4 (5.1)	1 (1.6)	4 (4.7)
***PDGFRA***	57 (11.2)	43 (12.6)	10 (12.8)	3 (4.8)	9 (10.6)
Exon 18 D842V	27 (5.3)	30 (8.8)	N.P.	N.P.	5 (5.9)
Other	30 (5.9)	13 (3.8)	N.P.	N.P.	4 (4.7)
**Non-*KIT*/non-*PDGFRA***	64 (12.6)	24 (7.0)	9 (11.5)	3 (4.8)	12 (14.1)
Other	0 (0)	1 (0.3) ^2^	0	0	0

ACOSOG: American College of Surgeons Oncology Group; N.P.: not provided; PDGFRA: platelet-derived growth factor receptor alpha; SSG: Scandinavian Sarcoma Group. ^1^ Forty-two patients excluded: no consent prior to randomization (*n* = 3); patient did not have GIST in central pathology review (*n* = 15); overt metastases at the time of study entry (*n* = 24). ^2^ Unspecified *KIT* mutation.

**Table 4:** Influence of adjuvant imatinib on survival in *KIT* and *PDGFRA* mutational subsets.

Mutation Type	Survival Benefit from Adjuvant Imatinib	Comment
*Kit*		
Exon 11		
Deletion/indel	Substantial	66% of deaths avoided with 3 yr imatinib [45]
Substitution	Probable but not demonstrated	Benefit likely less than with deletion/indel mutations [37,44,45]
Insertion/duplication	Probable but not demonstrated	Sensitive to imatinib in vitro [37,44,45]
Exon 9	Unknown, but possible	Only an imatinib dose of 400 mg/day has been studied [37,44,45]
*PDGFRA*		
D842V	No benefit	Recurrence infrequent; imatinib-resistant [23]
Other	Probable but not demonstrated	Most imatinib-sensitive [44]
Non-*KIT*/non-*PDGFRA*	No benefit	*KIT*/*PDGFRA* mutation sometimes missed in testing [46]

## Data Availability

The data presented in this study are available in this article.
